# The Action of the Tincture of Iodine upon the Uterine Neck

**Published:** 1878-11

**Authors:** 


THE ACTION OF THE TINCTURE OF IODINE UPON THE UTERINE NECK.—The tincture of iodine does not act the same upon the healthy neck and on the diseased one. If the tissues of the neck are healthy, the coloration produced by its application is a uniform dark brown; if there is the least ulceration, there is produced a yellowish coloration, in contrast to the brown of the neighboring parts. Every granulation or vegetation becomes yellow and not brown. After the application of the actual cautery, which M. Laboulbene strongly recommends for ulceration of the neck, the cauterized part only is made yellow by the tincture so long as it is not heated. After cure the neck gradually becomes brown on the application of the tincture.

If the neck is large, without being ulcerated, and if the tincture of iodine brings to view little yellowish spots, we must suspect a neoplasm, and expect in a short time to have ulceration of the places where the tissue is colored. It is easy to see the utility of this application in the diagnosis of uterine affections.—*La France Medicale.*

